# Biopsychosocial factors of quality of life among middle-aged adults living alone in South Korea: a secondary data analysis using the 2017 Korea Community Health Survey

**DOI:** 10.1186/s13690-024-01342-0

**Published:** 2024-07-18

**Authors:** Hyerang Kim, Eun Jung Bae, Yunkyung Choi, Heesook Son

**Affiliations:** 1 Department of Nursing Science, Howon University, 64 Howondae 3gil, Impi, Gunsan city, Jeollabuk-do 54508 Republic of Korea; 2 Department of Nursing, Ansan University, 155Ansan Daehak-ro, Sangnok-gu, Ansan-si, Gyeonggi-do 15328 Republic of Korea; 3 College of Nursing, Chonnam National University, 160 Baekseo-ro, Dong-gu, Gwangju, 61469 Republic of Korea; 4 Red Cross College of Nursing, Chung-Ang University, 84 Heukseok-ro, Dongjak-gu, Seoul, 06974 Republic of Korea

**Keywords:** Quality of life, Middle-aged adults, Living alone, Biopsychosocial, Dynamic model

## Abstract

**Background:**

This study aimed to investigate quality of life (QoL) in middle-aged adults living alone and identify comprehensive biological, psychological, interpersonal, and contextual factors related to QoL using the dynamic biopsychosocial model. As a secondary analysis, this study used data from the 2017 Korea Community Health Survey conducted by the Korea Disease Control and Prevention Agency.

**Methods:**

Among the total 228,381 respondents, 10,639 middle-aged individuals aged 40–64 years from single-person households (5,036 men and 5,603 women) were included in the analysis. QoL was measured using the EuroQoL-5 Dimension (EQ-5D). The EQ-5D descriptive statistics were provided according to biological, psychological, interpersonal, and contextual factors. Considering the data structure of the multistage stratified cluster sampling method, a complex samples general linear model statistic was used to identify the predictors of QoL.

**Results:**

QoL was lower in those who had undesirable psychological status (e.g., more depressive symptoms, poor subjective health, and higher perceived stress), less engagement in social networking (less frequent contact with friends and less frequent participation in social activities such as religious activities, friendship gathering, and leisure), and lower physical, behavioral, and socioeconomic factors.

**Conclusions:**

This study’s findings indicate that psychological and interpersonal factors should be addressed and prioritized to improve the QoL of middle-aged adults living alone. By providing many opportunities for easily accessible social activities that meet the needs and interests of this demographic, their QoL can be improved through strengthening social support.

**Table:** 

Text box 1. Contributions to the Literature
• The psychological and interpersonal domains accounted for the largest portion of the variance in QoL, suggesting that interventions for these factors should be prioritized to improve the QoL of middle-aged adults living alone.
• For interpersonal factors, contact with friends and participation in interactive activities may significantly influence QoL.
• QoL for middle-aged adults living alone can be improved through strengthening social support.

## Background

Single-person households have been increasing worldwide. In South Korea, the proportion of single-person households has increased continuously, from 15.5% in 2000 to 29.3% in 2018 [1]. The proportion of single-person households is projected to reach 37.3% by 2047, with 40.5% comprising individuals aged 70 years and older [2]. In 2018, individuals in their 40–60 s, 20–30 s, and 70s or older comprised 46.4%, 34.4%, and 18.3% of all single-person households, respectively [1]. This indicated that middle-aged adults living alone accounted for the highest proportion of these households. This has been influenced by various factors, including unstable employment, increasing age at the time of first marriage, increasing divorce rates, split households, and separation due to children’s education [3, 4].

Although the definition of middle age remains controversial, most studies consider it to be the period of life between the ages of 40 and 65 years [5, 6]. Middle age is generally when physical degeneration begins, and socioeconomic stability is achieved [7]. Moreover, as a period of influx prior to old age, it is a prerequisite for healthy aging [8, 9]. Thus, a physically, mentally, and socially healthy middle-aged period is highly likely to lead to a healthy life in old age [10].

Quality of life (QoL) refers to the subjective wellbeing and satisfaction individuals experience. It can be explained as one’s health level based on physical, psychological, and social perspectives according to personal beliefs, experiences, and cognitive levels [11]. QoL can be described using various terms such as joy, subjective happiness, life satisfaction, psychological comfort, subjective wellbeing, life satisfaction, and psychological wellbeing [11, 12]. QoL is lower for older and middle-aged adults living alone than for those in multi-person households [13, 14]. Previous studies have shown that QoL is associated with physical factors such as age, sex, and the presence or absence of disease [15, 16]; psychological factors such as depression, stress, and subjective health status [17–19]; lifestyle and health behaviors [15, 20]; social relationships [20]; and environmental context [15, 21, 22].

QoL is a multidimensional concept; thus, a comprehensive approach is needed to identify relevant factors. Few studies have applied a systematic model to examine QoL in single-person households, and little is known regarding the QoL of middle-aged adults living alone. The dynamic biopsychosocial (DBPS) model comprehensively describes health based on biological, psychological, interpersonal, and contextual factors [23]. Therefore, the DBPS model is beneficial for explaining factors related to QoL in middle-aged single-person households. This study aimed to investigate QoL in this demographic and identify comprehensive biological, psychological, interpersonal, and contextual factors related to QoL using the DBPS model.

## Methods

### Study population

The study used secondary national data from the 2017 Korea Community Health Survey performed by the Korea Disease Control and Prevention Agency, collected from August 16 to October 31, 2017. The Korea Community Health Survey is conducted annually with adults aged 19 years or older to examine the health status of residents under the Community Health Act [24]. The survey employed a multistage, stratified, and random sampling method to represent the Korean population.

The primary target population of this study was middle-aged adults living alone. Among the total 228,381 respondents, 10,639 middle-aged individuals (40–64 years of age) from single-person households (5,036 men and 5,603 women) were included in the analysis.

### Theoretical framework

**Fig. 1:**
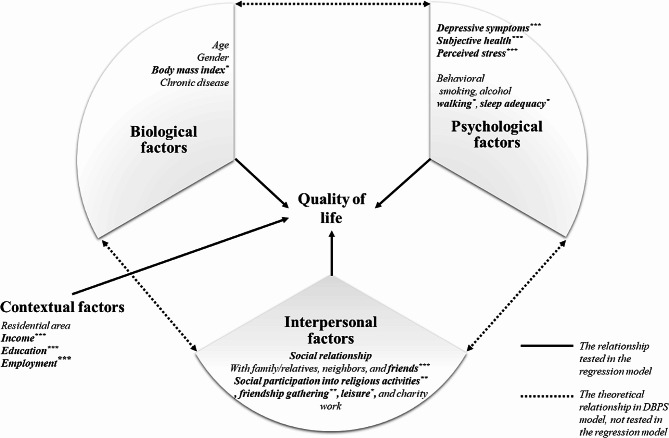
Conceptual framework of the dynamic biopsychosocial determinants of quality of life. ^*^p < 0.05, ^**^p < 0.01, ^***^p < 0.001

The DBPS model was developed based on general systems theory and ecological theory by applying ecological perspectives to multidimensional health characteristics [23]. The DBPS model explains health-related factors by dividing them into biological, psychological, and social categories. The social category includes both interpersonal and contextual factors. The factors that influence health are dynamically configured as a set, change continuously over time, and interact with each other. This model offers a multidimensional approach to health and has been applied in various fields [25–27]. Figure 1 depicts the conceptual framework of the DBPS determinants of QoL in middle-aged adults living in single-person households.

### Outcome variables: quality of life

QoL was measured using the EuroQoL-5 Dimension (EQ-5D) [28] to evaluate a standardized basic health index and determine overall health [29, 30]. The EQ-5D measures the following five items: mobility, self-care, usual activity, pain/disability, and anxiety/depression. Each item on current health status was rated on a 3-point scale: 1 point for “no problems,” 2 points for “some problems,” and 3 points for “severe problems.” The total score for responses of “some problems” or “serious problems” was calculated using the weighted QoL formula proposed by the Korea Centers for Disease Control and Prevention: EQ-5D index = 1 − (0.0081 + 0.1140*M2 + 0.6274*M3 + 0.0572*SC2 + 0.2073*SC3 + 0.0615*UA2 + 0.2812*UA3 + 0.0581*PD2 + 0.2353*PD3 + 0.0675*AD2 + 0.2351*AD3). A score closer to 1 indicates a more complete health status [31]. The Korean version of the EQ-5D has been found to be reliable and valid in previous studies [32]. Cronbach’s alpha was 0.757 in this study.

### Predictors of quality of life

Biological factors included age, sex, obesity as determined by height and weight, and the presence or absence of chronic diseases, such as hypertension and diabetes. Obesity was reclassified according to body mass index (BMI), determined by calculating “weight (kg)/height (m^2^)” based on an individual’s measured height and weight. A BMI of < 23.0 kg/m^2^ was classified as “normal,” 23–24.9 kg/m^2^ as “overweight,” and ≥ 25.0 kg/m^2^ as “obesity” [33].

Psychological factors were measured based on depressive symptoms, subjective health levels, subjective stress levels, and health-related behavioral factors such as current smoking, drinking, exercise, and sleep hours. Depressive symptoms were assessed using the Patient Health Questionnaire-9, with total scores ranging from 0 to 27 points. Depression severity was categorized into two groups: minimal to mild (~ 9 points) and moderate to severe (10 ~ 27 points). Cronbach’s alpha was 0.834 in this study.

Subjective health levels were measured on a 5-point scale ranging from 1 point for “very good” to 5 points for “very bad” in response to the question: “How do you usually think of your health?” This was used to determine respondents’ health status––the higher the score, the higher the subjective health.

Subjective stress levels were measured to assess stress levels in daily life (study, work, and housework) based on responses to the following question: “How much stress do you usually feel in your daily life?” This item was measured on a scale ranging from 1 point for “I feel very stressed” to 4 points for “I hardly feel stressed.” Higher scores indicated higher subjective stress levels.

Current smoking status was measured by asking participants about current (“yes”) versus past (but not current) smoking habits and never smoking at all (“no”). Current drinking status was measured by asking participants whether they engaged in binge drinking. Regular walking was defined as walking at least 5 days per week for at least 20 min per day. Sleep adequacy was defined as 7–8 h of sleep per day.

Interpersonal factors were measured based on respondents’ social networks and participation in social activities. Social networks were assessed to determine the frequency of contact with relatives, family members, neighbors, and friends who were available for direct two-way communication in person or by telephone. The respondents were asked to list the person they contacted most frequently, which was measured as an ordinal variable: less than once a month, once a month, 2–3 times a month, once a week, 2–3 times a week, and more than 4 times a week. For the analysis, this variable was recoded as < once/week, 1–3 times/week, and ≥ 4 times/week.

Social activities included religious activities, friendship gatherings, leisure activities, and charitable work. Respondents were asked to answer “yes” or “no” to the following question: “Do you regularly participate in each category of social activities at least once per month?”

Contextual factors included residence, monthly household income, education level, employment, and marital status. Residential areas were divided into urban and rural. Monthly household income was measured as the average monthly household income over the past year. Based on the 2017 median income of single-person households (KRW 1,652,931), single-person household income was classified into two groups: < KRW 2 million and ≥ KRW 2 million. Education level was reclassified as a high school diploma or higher, and employment status was recorded as currently employed or unemployed. Marital status was recorded as married, divorced, separated, widowed, or never married.

### Statistical analysis

Considering the data structure of the multistage stratified cluster sampling method used in the Korea Community Health Survey, a complex samples statistic was used for the analysis. Complex samples descriptives and crosstabs statistics were used to examine the differences in the score of EQ-5D by each variable of DBPS factors. A complex samples general linear model was used to determine the predictors of QoL by biological, psychological, behavioral, interpersonal, and contextual factors. The predicting model was estimated again for the DBPS model, using all potential predictors from the biological, psychological, behavioral, interpersonal, and contextual factors. Unstandardized b coefficients (B) with 95% confidence intervals (95% CI) were estimated. A *p*-value < 0.05 indicated statistical significance. All statistical analyses were performed using IBM SPSS version 29.0 (New York, NY, USA).

## Results

Among middle-aged adults living alone, QoL scores differed across biological, psychological, behavioral, interpersonal, and contextual factors (Table 1). In the biological domain, QoL scores were lower among those who were older (*p* < 0.001), female (*p* = 0.036), had obesity (*p* = 0.004), and had diabetes (*p* < 0.001). In the psychological domain, individuals with more severe depressive symptoms (*p* < 0.001), poorer subjective health (*p* < 0.001), and greater perceived stress (*p* < 0.001) had lower QoL scores. In the behavioral domain, QoL scores were lower among those who were current smokers (*p* = 0.016), did not walk regularly (*p* < 0.001), and lacked sleep adequacy (*p* < 0.001). Regarding social relationships, individuals who met with family or friends less than once per week had lower QoL scores than those with more frequent contact (*p* < 0.001). QoL scores differed by social participation––it was higher in those who participated in friendship gatherings (*p* < 0.001), leisure activities (*p* < 0.001), or charitable work (*p* < 0.001). Unlike other social participation, those who did not participate in religious activities had lower QoL scores (*p* = 0.024). In the contextual domain, QoL scores were lower among those who were living in urban areas (*p* < 0.001), had lower incomes (*p* < 0.001), had lower education levels (*p* < 0.001), and were unemployed (*p* < 0.001). Additionally, QoL scores were lower for individuals who were divorced, widowed, or never married (*p* < 0.001).

**Table 1:** Quality of life in the dynamic biopsychosocial model (*N* = 10,639)

Variables	Quality of life (EQ-5D)		
	n (%)	Mean ± SE	*p-value*
Biological			
Age, years			
40–49	1816(22.5)	0.91 ± 0.01	< 0.001
50–59	5236(51.8)	0.87 ± 0.01	
60–64	3587(25.7)	0.82 ± 0.01	
Sex			
Male	5036(50.5)	0.87 ± 0.01	0.036
Female	5603(49.5)	0.86 ± 0.00	
BMI, kg/m^2^			
Underweight/normal (< 23.0)	4157(41.6)	0.88 ± 0.01	0.004
Overweight (23.0–24.9)	2562(24.9)	0.89 ± 0.01	
Obesity (≥ 25.0)	3472(33.5)	0.86 ± 0.01	
Chronic disease			
None	7185(70.0)	0.89 ± 0.01	< 0.001
Diabetes	1308(10.9)	0.75 ± 0.01	
Hypertension	2142(19.1)	0.84 ± 0.01	
Psychological			
Depressive symptoms			
Minimal to mild	10,108(91.8)	0.89 ± 0.00	< 0.001
Moderate to severe	494(5.2)	0.38 ± 0.04	
Subjective health			
Very poor/poor	2039(20.4)	0.63 ± 0.01	< 0.001
Moderate	5037(48.3)	0.91 ± 0.00	
Good/very good	3291(31.2)	0.96 ± 0.00	
Perceived stress			
No	8142(74.5)	0.91 ± 0.00	< 0.001
Yes	2497(25.5)	0.75 ± 0.01	
Behavioral			
Smoking			
Past smoking/non-smoking	7712(71.4)	0.87 ± 0.00	0.016
Current smoking	2916(28.6)	0.85 ± 0.01	
Drinking			
Abstainer/moderate drinker	7090(79.2)	0.88 ± 0.00	0.339
Binge drinker	1724(20.8)	0.89 ± 0.01	
Walking			
Regular walking	4715(47.9)	0.89 ± 0.00	< 0.001
Irregular walking	5922(52.1)	0.84 ± 0.01	
Sleep adequacy			
Adequate	4774(43.7)	0.90 ± 0.00	< 0.001
Inadequate	5862(56.3)	0.84 ± 0.01	
Interpersonal			
Social relationship			
Family/relatives			
< once/week	4,775(50.5)	0.84 ± 0.01	< 0.001
1–3 times/week	3,005(26.4)	0.89 ± 0.01	
≥ 4 times/week	2,852(23.1)	0.89 ± 0.01	
Neighbors			
< once/week	5,074(62.0)	0.87 ± 0.01	0.839
1–3 times/week	2,299(18.6)	0.87 ± 0.01	
≥ 4 times/week	3,194(19.4)	0.87 ± 0.01	
Friends			
< once/week	4,764(46.9)	0.83 ± 0.01	< 0.001
1–3 times/week	3,178(30.4)	0.89 ± 0.01	
≥ 4 times/week	2,653(22.7)	0.91 ± 0.00	
Social participation			
Religious			
No	7,755(73.2)	0.87 ± 0.00	0.024
Yes	2,882(26.8)	0.85 ± 0.01	
Friendship gatherings			
No	4,752(45.9)	0.81 ± 0.01	< 0.001
Yes	5,886(54.1)	0.92 ± 0.00	
Leisure			
No	7,539(67.5)	0.84 ± 0.01	< 0.001
Yes	3,099(32.5)	0.93 ± 0.00	
Charity work			
No	9,603(90.8)	0.86 ± 0.00	< 0.001
Yes	1,034(9.2)	0.92 ± 0.01	
Contextual			
Residential area			
Urban	5892(78.8)	0.86 ± 0.00	< 0.001
Rural	4747(21.2)	0.89 ± 0.01	
Income (per month)			
< 2,000,000 KRW	5044(41.3)	0.77 ± 0.01	< 0.001
≥ 2,000,000 KRW	5256(58.7)	0.93 ± 0.00	
Education			
< High school graduate	3739(27.5)	0.79 ± 0.01	< 0.001
≥ High school graduate	6872(72.5)	0.90 ± 0.00	
Employment			
Unemployed	3085(30.6)	0.73 ± 0.01	< 0.001
Employed	7545(69.4)	0.93 ± 0.00	
Marital status			
Married	2,550(27.0)	0.93 ± 0.00	< 0.001
Divorced	3,047(30.2)	0.82 ± 0.01	
Separated	932(7.9)	0.92 ± 0.01	
Widowed	2,109(14.1)	0.84 ± 0.01	
Never married	1,929(20.8)	0.85 ± 0.01	

BMI: body mass index; CI: confidence interval; EQ-5D: EuroQoL-5 Dimension; KRW: Korean Won

Table 2 shows the significant predictors of QoL for each domain of the DBPS model. The significant predictors among biological variables were age, sex, and chronic diseases such as diabetes and hypertension. These explained 4.1% of the variance (Wald F = 51.416, R^2^ = 0.041, *p* < 0.001). QoL was lower for those who were older (B [95% CI] = -0.004 [-0.005, -0.003], *p* < 0.001), female (B [95% CI] = 0.021 [0.009, 0.033] for men, *p* < 0.001), and had chronic diseases like diabetes and hypertension (B [95% CI] = -0.177 [-0.144, -0.090] for diabetes; B [95% CI] = -0.050 [-0.068, -0.033] for hypertension; *p* < 0.001 for both). The model for the psychological domain explained 27.2% of the variance (Wald F = 240.089, R^2^ = 0.272, *p* < 0.001) and had the highest R^2^ among all domains of the DBPS model. QoL was lower for those with more depressive symptoms (B [95% CI] = 0.373 [0.302, 0.443] for minimal to mild depressive symptoms; *p* < 0.001), poorer subjective health (B [95% CI] = -0.247 [-0.268, -0.225] for very poor/poor; B [95% CI] = -0.033 [-0.041, -0.025] for moderate; *p* < 0.001 for both), and higher perceived stress (B [95% CI] = 0.064 [0.048, 0.080] for “not stressful,” *p* < 0.001). Variables in the behavioral domain significantly predicted QoL (Wald F = 22.362, R^2^ = 0.017, *p* < 0.001). QoL was lower in those who were current smokers (B [95% CI] = 0.028 [0.010, 0.045] for past smoker/non-smoker, *p* = 0.002), abstained from alcohol or were moderate drinkers (B [95% CI] = -0.019 [-0.037, -0.002], *p* = 0.029), did not walk regularly (B [95% CI] = 0.044 [0.030, 0.058] for regular walks, *p* < 0.001), and lacked adequate sleep (B [95% CI] = 0.047 [0.033, 0.661] for adequate, *p* < 0.001). In the interpersonal domain, QoL was lower among those who participated less frequently in social networking (*p* < 0.001) and social activities (*p* < 0.001). In the contextual domain, QoL was lower for individuals who lived in urban areas (*p* < 0.001), had lower incomes *p* < 0.001), had lower education levels (*p* < 0.001), and were unemployed (*p* < 0.001) (Wald F = 83.647, R^2^ = 0.148, *p* < 0.001). QoL scores were higher for individuals who were separated (*p* < 0.001) or widowed (*p* < 0.001) versus those who were never married.

**Table 2:** Complex samples general linear model for quality of life according to each domain of the dynamic biopsychosocial model

Predictors	B[95% CI]	*p-value*	Model fit
Biological			
Age, years	-0.004[-0.005, -0.003]	< 0.001	Wald F = 51.416
Sex			R^2^ = 0.041
Male	0.021[0.009, 0.033]	< 0.001	*p* < 0.001
Female (ref.)			
BMI, kg/m^2^	-0.001[-0.002, 0.000]	0.088	
Chronic disease			
None (ref.)			
Diabetes	-0.177[-0.144, -0.090]	< 0.001	
Hypertension	-0.050[-0.068, -0.033]	< 0.001	
Psychological			
Depressive symptoms			Wald F = 240.089 R^2^ = 0.272 *p* < 0.001
Minimal to mild	0.373[0.302, 0.443]	< 0.001	
Moderate to severe (ref.)			
Subjective health			
Very poor/poor	-0.247[-0.268, -0.225]	< 0.001	
Moderate	-0.033[-0.041, -0.025]	< 0.001	
Good/very good (ref.)			
Perceived stress			
No	0.064[0.048, 0.080]	< 0.001	
Yes (ref.)			
Behavioral			
Smoking			Wald F = 22.362
Past smoking/non-smoking	0.028[0.010, 0.045]	0.002	R^2^ = 0.017
Current smoking (ref.)			*p* < 0.001
Drinking			
Abstainer/moderate drinker	-0.019[-0.037, -0.002]	0.029	
Binge drinker (ref.)			
Walking			
Regular walking	0.044[0.030, 0.058]	< 0.001	
Irregular walking (ref.)			
Sleep adequacy			
Adequate	0.047[0.033, 0.661]	< 0.001	
Inadequate (ref.)			
Interpersonal			
Social relationships			Wald F = 40.024 R^2^ = 0.056 *p* < 0.001
Family/relatives			
< once/week	-0.026[-0.041, -0.011]	0.001	
1–3 times/week	0.002[-0.016, 0.021]	0.793	
≥ 4 times/week (ref.)			
Neighbors			
< once/week	0.021[-0.001, 0.043]	0.058	
1–3 times/week	0.010[-0.012, 0.031]	0.395	
≥ 4 times/week (ref.)			
Friends			
< once/week	-0.040[-0.054, -0.025]	< 0.001	
1–3 times/week	-0.015[-0.030, 0.000]	0.053	
≥ 4 times/week (ref.)			
Social participation			
Religious			
No	0.031[0.014, 0.048]	< 0.001	
Yes (ref.)			
Friendship gathering			
No	-0.086[-0.101, 0.072]	< 0.001	
Yes (ref.)			
Leisure			
No	-0.058[-0.068, -0.048]	< 0.001	
Yes (ref.)			
Charity work			
No	-0.016[-0.031, -0.002]	0.028	
Yes (ref.)			
Contextual			
Residential area			Wald F = 83.647
Urban	-0.023[-0.033, -0.013]	< 0.001	R^2^ = 0.148
Rural (ref.)			*p* < 0.001
Income (per month)			
< 2,000,000 KRW	-0.078[-0.092, -0.065]	< 0.001	
≥ 2,000,000 KRW (ref.)			
Education			
< High school graduate	-0.055[-0.072, -0.037]	< 0.001	
≥ High school graduate (ref.)			
Employment			
Unemployed	-0.161[-0.180, -0.142]	< 0.001	
Employed (ref.)			
Marital status			
Married	0.054[0.07, 0.070]	< 0.001	
Divorced	-0.010[-0.032, 0.012]	0.364	
Separated	0.056[0.037, 0.075]	< 0.001	
Widowed	0.040[0.017, 0.062]	< 0.001	
Never married (ref)			

BMI: body mass index; CI: confidence interval; KRW: Korean Won

Table 3 presents the predictive factors for QoL when all potential variables from each domain of the DBPS model were evaluated. QoL was not associated with any variables of the biological domain, considering the simultaneous influences of other DBPS model variables. All psychological variables were significantly associated with QoL. Specifically, it was lower among those who had more depressive symptoms (B [95% CI] = 0.255 [0.202, 0.307], *p* < 0.001), poorer subjective health (B [95% CI] = -0.163 [-0.184, -0.142] for very poor/poor; (B [95% CI] = -0.015 [-0.022, -0.008] for moderate; *p* < 0.001), and higher perceived stress (B [95% CI] = 0.063 [0.048, 0.077], *p* < 0.001). In the behavioral domain, regular walking (*p* = 0.021) and sleep adequacy (*p* = 0.005) were significantly associated with QoL. Among interpersonal variables, engagement in social networking and participation in social activities were significant predictors for QoL. Interestingly, regarding social relationships, contact with friends less than once a week was negatively associated with QoL (B [95% CI] = 0.021 [-0.033, -0.009], *p* < 0.001), while contact 1–3 times a week was positively associated with QoL (B [95% CI] = -0.018 [-0.029, -0.007], *p* = 0.001) compared with more than 4 times a week.

**Table 3:** Complex samples general linear model for quality of life based on the dynamic biopsychosocial model

Predictors		B[95% CI]			*p-value*
Biological					
Age, years		-0.001[-0.002, 0.000]			0.249
Sex					
Male		0.001[-0.011, 0.014]			0.823
Female (ref.)					
BMI, kg/m^2^		-0.001[-0.002, 0.000]			0.052
Chronic disease					
None (ref.)					
Diabetes		-0.010[-0.027, 0.007]			0.247
Hypertension		-0.013[-0.029, 0.003]			0.104
Psychological					
Depressive symptoms					
Minimal to mild		0.255[0.202, 0.307]			< 0.001
Moderate to severe (ref.)					
Subjective health					
Very poor/poor		-0.163[-0.184, -0.142]			< 0.001
Moderate		-0.015[-0.022, -0.008]			< 0.001
Good/very good (ref.)					
Perceived stress					
No		0.063[0.048, 0.077]			< 0.001
Yes (ref.)					
Behavioral					
Smoking					
Past smoking/non-smoking		0.009[-0.003, 0.021]			0.126
Current smoking (ref.)					
Drinking					
Abstainer/moderate drinker		-0.003[-0.016, 0.011]			0.700
Binge drinker (ref.)					
Walking					
Regular walking		0.011[0.002, 0.020]			0.021
Irregular walking (ref.)					
Sleep adequacy					
Adequate		0.014[0.004, 0.024]			0.005
Inadequate (ref.)					
Interpersonal					
Social relationship					
Family/relatives					
< once/week		-0.005[-0.017, 0.007]			0.404
1–3 times/week		0.005[-0.007, 0.017]			0.412
≥ 4 times/week (ref.)					
Neighbors					
< once/week		-0.002[-0.021, 0.018]			0.860
1–3 times/week		-0.001[-0.020, 0.017]			0.902
≥ 4 times/week (ref.)					
Friends					
< once/week		-0.021[-0.033, -0.009]			< 0.001
1–3 times/week		-0.018[-0.029, -0.007]			0.001
≥ 4 times/week (ref.)					
Social participation					
Religious					
No		0.017[0.006, 0.028]			0.002
Yes (ref.)					
Friendship gathering					
No		-0.013[-0.022, -0.004]			0.007
Yes (ref.)					
Leisure					
No		-0.009[-0.017, -0.000]			0.040
Yes (ref.)					
Charity work					
No		-0.012[-0.027, 0.003]			0.116
Yes (ref.)					
Contextual					
Residential area					
Urban		-0.009[-0.019, 0.002]			0.103
Rural (ref.)					
Income (per month)					
< 2,000,000 KRW		-0.025[-0.035, -0.015]			< 0.001
≥ 2,000,000 KRW (ref.)					
Education					
< High school graduate		-0.028[-0.043, -0.012]			< 0.001
≥ High school graduate (ref.)					
Employment					
Unemployed		-0.077[-0.091, -0.062]			< 0.001
Employed (ref.)					
Marital status					
Married		0.017[0.004, 0.029]			0.009
Divorced		-0.009[-0.026, 0.008]			0.293
Separated		0.015[-0.014, 0.023]			0.641
Widowed		0.004[0.000, 0.030]			0.056
Never married (ref)					
		Wald F = 40.518			
		R^2^ = 0.330			
		*p* < 0.001			

BMI: body mass index; CI: confidence interval; KRW: Korean Won

Regarding social participation, compared with participation in friendship gatherings and leisure activities as a reference value, non-participation in these social activities was negatively associated with QoL (friendship gatherings: B [95% CI] = -0.013 [-0.022, -0.004], *p* = 0.007; leisure: B [95% CI] = -0.009 [-0.017, -0.000], *p* = 0.040). However, participation in religious activities was positively associated with QoL (B [95% CI] = 0.017 [0.006, 0.028], *p* = 0.002**)**. In the contextual domain, income and education were significant predictors of QoL. Specifically, both were negatively associated with QoL (income: B [95% CI] = -0.025 [-0.035, -0.015], *p* < 0.001; education: B [95% CI] = -0.028 [-0.043, -0.012], *p* < 0.001). Finally, compared with those who were never married, being currently married was positively associated with QoL (B [95% CI] = 0.017[0.004, 0.029], *p* = 0.009).

## Discussion

Single-person households have been increasing worldwide, with middle-aged adults living alone comprising the highest proportion. To experience a healthy old age, middle-aged adults must maintain or improve their physical and psychosocial health. Considering that QoL is multidimensional, the current study applied a comprehensive approach using the DBPS model to examine the QoL of the target population.

We first performed multiple regression analyses to determine the influence of each domain of the DBPS model on QoL. The psychosocial domain accounted for the largest portion of the QoL variance, suggesting that interventions for psychological factors should be prioritized to improve the QoL of middle-aged adults living alone.

Our findings on the negative influence of depressive symptoms on QoL in this demographic are consistent with and extend those of previous studies [17, 18]. QoL is well known to correlate with symptoms of all major psychiatric disorders, and depressive symptoms are strong predictors of QoL relative to other psychopathologies [34]. Moreover, many researchers have expressed concerns regarding the high prevalence of depression and general mental health disorders among those living in single-person households [35, 36]. A study examining the long-term effects of depressive symptoms reported that depressive symptoms in middle-aged adults independently predicted the development of limitations in basic activities of daily living and mobility in older adults [37]. Consequently, addressing depressive symptoms in middle-aged adults living alone may present an opportunity to improve QoL in this population as well as in older adults by reducing the risk of losing functional independence.

As supported by our study’s findings, subjective health is significantly associated with QoL in middle-aged adults [38]. QoL shares several attributes with subjective health––it is a broad concept that incorporates multidimensional features, including individual physical, psychological, and social health [39]. In this context, subjective health status is a major contributor to QoL [38, 40]. In particular, a previous study found that middle-aged individuals from single-person households had poorer subjective health than those living with others [41]. This emphasizes that improving the subjective health of middle-aged adults living alone is essential to improving their QoL. Pasanen et al. [40] conducted a more detailed investigation into subjective health among adults living alone by identifying subjective health profiles among this population. The characteristics of each profile group showed that this population is heterogeneous in terms of subjective health. Accordingly, additional studies identifying potential subjective health profiles among middle-aged adults living alone could help develop more specific strategies for improving QoL in this population.

Consistent with a previous study [19], our findings indicate that perceived stress highly correlates with QoL in middle-aged adults living alone. Stress is a well-known predictor of depressive symptoms. A recent cohort study reported that daily life was more stressful for middle-aged adults than people in other life stages [42]. Moreover, high perceived stress in midlife results from middle age representing a period that is instrumental in the success and development of others in the family, at work, and in society [42]. People experience stress from many sources, ranging from mundane occurrences to life events that require significant individual adjustment [43]. Stressors may differ between middle-aged people living alone and multi-person households. Additional studies should be performed to identify the specific stressors in middle-aged adults living alone. These findings can guide these individuals to improve their QoL by developing strategies to cope with different types of stressors.

In this study, contextual factors, including socioeconomic status (SES), explained 13.8% of the variance in QoL among middle-aged adults living alone. The close association between SES and QoL has been extensively documented in the literature [21, 22]. SES is correlated with various factors associated with QoL. For example, a higher income may provide better nutrition, housing, and recreation. Moreover, education is a fundamental factor in shaping earnings potential. Further, employment allows people to gain various resources to improve their lives [44, 45]. In this context, SES is a major factor in determining QoL for middle-aged adults living alone and the general population. Studies on this demographic have suggested a pathway through which SES could affect QoL by linking to factors associated with QoL [46, 47]. For example, Choi and Lee [46] reported that SES significantly positively affects subjective health and identified social network satisfaction and self-esteem as significant negative mediators of the relationship between SES and QoL. As this study revealed, subjective health and depressive symptoms are significant contributors to QoL among middle-aged adults living alone. SES has characteristics that cannot be easily modified. Therefore, when establishing interventions to improve QoL in this demographic, it may be more helpful to explore the mechanisms leading to QoL according to SES rather than focusing on changing an individual’s SES. Improvements in QoL can be expected by identifying and strengthening factors that can mitigate the negative impact of low SES on QoL.

We also investigated the influence of social relationships on QoL among middle-aged adults living alone. We hypothesized that the frequency of contact with family, neighbors, and friends would correlate with QoL in this population. Social relationships play an important role in the subjective wellbeing of adults living alone by providing them with support and reducing their social isolation [48–50]. People receive tangible or psychological support from others through their personal networks [50, 51]. Previous studies have reported that having a good network of friends and relatives is positively associated with wellbeing among adults living alone [52, 53]. Although the frequency of contact with family and neighbors was not correlated with QoL in our study, we found that middle-aged adults living alone with more contact with friends had higher QoL. This partially supports the conclusions of previous studies. A study examining the quality of the personal networks of people who live alone found that, compared with people living with their families, those living alone felt less close to their relatives and contacted friends and acquaintances more frequently [50]. In this context, even for middle-aged adults living alone, contact with friends may have a more significant influence on QoL than contact with other groups. Social relationships should be evaluated more broadly by including the frequency of contact with others, social network size, relationship quality, and satisfaction [50, 53]. This study could not include these variables because of its design as a secondary data analysis. Nonetheless, further research should examine more comprehensive assessments of social relationships and their link to QoL in middle-aged adults living alone.

Our study also found a positive influence of participation in various social activities on the QoL of the target demographic, which aligns with previous studies’ findings [54]. A longitudinal study of middle-aged adults aged 50–59 years reported that psychological distress was more strongly correlated with a lack of social participation than with living alone and social participation can increase QoL by strengthening social support through expanding social networks [55]. In this context, the social participation of middle-aged adults living alone may be significantly related to their QoL. Therefore, this demographic should be provided with easily accessible opportunities for social activities that meet their needs and interests.

Our results regarding the relationship between health behaviors and QoL were somewhat mixed compared with the results of previous studies by gender. In a previous study of adult men [20], alcohol consumption was not correlated with QoL. By contrast, among young and middle-aged women, alcohol consumption was associated with improvements in physical health-related QoL after two years but not associated with mental health-related QoL. These gender differences were also found in the association between smoking and QoL in adults: smoking was associated with lower QoL in men but not in women [15]. Studies on the relationship between health behaviors and QoL among middle-aged adults living alone are limited. To specifically identify the effects of health behaviors on the QoL of the target population, additional research should be performed to examine how the effects of smoking and alcohol consumption, which are considered major risky health behaviors, differ according to gender.

No significant relationship was found in this study between the presence or absence of diabetes and hypertension, which are representative chronic diseases, and QoL. Previous studies have reported an inverse association between the number of chronic diseases and QoL among older adults [55, 56]. These results may be related to increased multimorbidity in older adults, longer disease duration, and subsequent activity restriction [57]. To date, few studies have examined the effects of chronic diseases on the QoL of middle-aged adults living alone. Further studies that consider illness duration and severity are needed to clarify the impact of chronic diseases on QoL among this population.

The current study provides a large dataset of community-dwelling middle-aged adults living alone, which is potentially useful for investigating factors that may influence QoL. However, our study also has several limitations. First, data constraints due to the use of secondary data are a severe limitation. Middle-aged adults living alone represent a heterogeneous group. For example, they can be divided into different groups based on the timing of their transition to living alone, length and frequency of living alone, family formation trajectories, and path to living alone [3]. Limited information on these characteristics prevented us from adequately controlling for the heterogeneity in this group. Second, the use of self-report measures creates the risk of potential bias. Although the EQ-5D is an important and widely used measure of QoL, it may have a high percentage of participants in the best health state due to ceiling effects [58]. Finally, because this was a cross-sectional study, any associations found between the QoL of middle-aged adults living alone and related factors do not imply a causal relationship. A prospective study is needed to further clarify the causal relationships between QoL and related factors.

## Conclusions

Given the rapid increase in the number of middle-aged single-person households, more attention should be paid to QoL in this population. This study fills a gap in the literature on the QoL of this demographic. The findings demonstrate that psychosocial factors should be addressed and prioritized to improve QoL among them. In addition, providing opportunities for easily accessible social activities that meet the needs and interests of middle-aged adults living alone could be a useful strategy to improve QoL in this population by strengthening social support.

## Data Availability

The data used in this study are available upon request from https://chs.kdca.go.kr/chs/main.do, accessed July 4, 2020.

## Abbreviations

BMI: body mass index
CI: confidence interval
DBPS: dynamic biopsychosocial
EQ-5D: EuroQoL-5 Dimension
QoL: Quality of life
SES: socioeconomic status
